# Report about term infants with severe hyperbilirubinemia undergoing exchange transfusion in Southwestern China during an 11-year period, from 2001 to 2011

**DOI:** 10.1371/journal.pone.0179550

**Published:** 2017-06-29

**Authors:** Canfeng Yu, Huifan Li, Qiannan Zhang, Huayun He, Xinhong Chen, Ziyu Hua

**Affiliations:** 1 Department of Neonatology, Children’s Hospital of Chongqing Medical University, Chongqing, China; 2 Ministry of Education Key Laboratory of Child Development and Disorders, Chongqing, China; 3 Key Laboratory of Pediatrics in Chongqing, Chongqing, China; 4 Chongqing International Science and Technology Cooperation Center for Child Development and Disorders, Chongqing, China; Laval University, CANADA

## Abstract

**Objectives:**

This study was intended to explore the etiology and risk factors of severe neonatal hyperbilirubinemia and to analyze the adverse events associated with ECT (Exchange Transfusion), as well as to identify the factors related to the poor prognosis.

**Methods:**

All of the full-term neonates who had undergone ECT for hyperbilirubinemia at Children’s Hospital of Chongqing Medical University from January 2001 to December 2011 were enrolled in this study. General demographic characteristics, comorbidities, pre- and post-exchange TSB(Total Serum Bilirubin) levels, duration and frequency of ECT, and clinical outcomes were recorded and analyzed anonymously.

**Results:**

Of 614 total infants, 368 patients (59.9%) with ABO incompatibility were identified, of whom 197 (53.5%) developed acute bilirubin encephalopathy (ABE) and 16 (4.3%) suffered a poor prognosis. The etiology was unidentified in 103 patients (16.8%), of whom 62 (60.1%) developed ABE and 9 (8.7%) had a poor prognosis. Identified adverse events secondary to ECT included thrombocytopenia (54.6%), hyperglycemia (42.8%), apnea (3.3%) and necrotizing enterocolitis (NEC) (1.3%). No ECT-related mortality was documented in this study.

**Conclusions:**

The etiology, peak TSB level before ECT, and time of ECT had a significant impact on the outcome of severe neonatal hyperbilirubinemia. ABO incompatibility was the most common cause of extreme neonatal hyperbilirubinemia. Pathological weight loss could be involved in the development of extreme hyperbilirubinemia with an unidentified cause.

## Introduction

Neonatal hyperbilirubinemia remains a common disease leading to hospital admission during the neonatal period. Severe hyperbilirubinemic neonates could develop acute bilirubin encephalopathy or permanent neurological sequelae, kernicterus, which is characterized by cerebral palsy, impaired mental development, and neurological deafness. According to a survey of 33 nationwide hospitals launched by the neonatal group of the pediatric branch of the Chinese Medical Association, there were a total of 348 newborns that suffered hyperbilirubinemia, which accounted for 4.8% of newborn patients [1]. Even in developed countries, there were still several reports of newborn deaths from hyperbilirubinemia or complications of ECT. Cheng SW *et al* [2] have reported on 413 newborns with hyperbilirubinemia in Taiwan (GA ≥34 weeks, TSB ≥20 mg/L) in the past 13 years, and 111 (26.9%) of them were extremely hyperbilirubinemic term newborns (TSB ≥25 mg/L). Palmer DC *et al* [3] have studied 41,057 jaundiced newborns (TSB ≥157 μmol/L) during 10 years: 4406 newborns (10.7%) suffered hyperbilirubinemia, 63 (1.4%) of whom died. Ebbesen F *et al* [4] have investigated 128,344 newborns at the approximate age of one month in Denmark over 2 years; exchange transfusion was indicated in 32 newborns, i.e., the morbidity rate of hyperbilirubinemia was 25/100,000.

However, so far, there have been few large sample studies on severe hyperbilirubinemic neonates in China. We accomplished this retrospective analysis on hyperbilirubinemic term infants referred to Children’s Hospital of Chongqing Medical University during 11 years (2001–2011), investigating the icterogenic causes and risk factors, discussing the ECT-related adverse effects, and analyzing the factors associated with the prognosis.

As the center of the Neonatal Emergency Transport System (NETS), Children’s Hospital of Chongqing Medical University annually receives 6,500 to 8,000 neonates, among whom 1,545 to 1,790 critically ill neonates are transferred from more than 50 hospitals. Especially after implementation of the synchronous exchange transfusion via double peripheral vessels in 2000, a host of extremely hyperbilirubinemic neonates from surrounding areas, such as Chongqing, Guizhou, and Yunnan, were transferred to this center for exchange transfusion. Therefore, the retrospective analysis of neonates who received ECT for severe hyperbilirubinemia in this center during an 11-year period (2001–2011) might reflect the status quo regarding the approach to treat extremely hyperbilirubinemic neonates in Southwestern China.

## Subjects and settings

We reviewed the medical records of all full-term neonates who received ECT (through peripheral vessels [5]) for hyperbilirubinemia at Children’s Hospital of Chongqing Medical University from January 2001 to December 2011, referring to the latest guidelines issued by the AAP [6][7]. Inclusion criteria were 37 weeks ≤ GA < 42 weeks and age on admission ≤168 h. Those without complete medical records were excluded. Finally, 614 newborns were enrolled in this study. Data were then recorded, including age on admission, onset time of jaundice, TSB levels before and after ECT, comorbidities, blood volume, time of ECT, cranial imaging results and clinical outcomes. Ethics approval for this study was obtained from the Institutional Review Board of Children's Hospital, Chongqing Medical University. Verbal consent was obtained from parents and guardians of participants.

## Definitions

1. ECT-related mortality was defined as neonatal death within 5 days after exchange transfusion that was directly related to the ECT procedure.

2. ABE mainly refers to acute clinical manifestations of bilirubin neurotoxicity observed in the first week after birth, whereas kernicterus [8][9] refers to chronic and permanent clinical sequelae of bilirubin neurotoxicity. In addition to the manifestations of hyperbilirubinemia, there are typical nervous system features, including drowsiness, convulsion, abnormal muscle tone and opisthotonos. MRIs of some newborn patients show characteristic symmetrical hypertensive signals, such as a change on the globus pallidus in T1 and T2 weighted images, or brain stem auditory evoked potential (BAEP) showing high frequency hearing loss.

3. The Neonatal Behavioral Neurological Assessment (NBNA) was established by Xiulan Bao in China, in reference to the Neonatal Behavioral Assessment Scale (NBAS) by Brazelton and the neuromotor measurement by Amiel-Tison. The NBNA assesses functional abilities, most reflexes and responses, and stability of behavioral status during examination. It consists of five clusters: behavior (six items), passive tone (four items), active tone (four items), primary reflexes (three items), and general assessment (three items). Each item includes three levels (0, 1 and 2). The maximum total score is 40. Infants with scores under 35 could be considered abnormal.

4. Clinical BIND score schema were provided by Johnson *et al*[9]. In terms of infants’ mental status, muscle tone and cry, we obtained a total score to grade the severity of ABE. The data were tabulated retrospectively, using a medical chart review. The progression of ABE was categorized as subtle, moderate or advanced.

5. ECT-related adverse events were defined as any complication which occurred within 5 days after exchange transfusion.

## Statistical analysis

All data were analyzed using *SPSS* 18.0 software for Windows. Measurement data were reported as the mean ± *standard deviation*. Counting data were reported as numbers of cases. A normality test was conducted, adopting the *Kolmogorov*-*Smirnov* test. Measurement data were analyzed using *t*-test if normally distributed, otherwise a *rank*-*sum* test was adopted. Counting data were analyzed using a *chi*-*squared* test. *P* values less than 0.05 were considered statistically significant.

## Results

### 1. General condition

**Fig 1:**
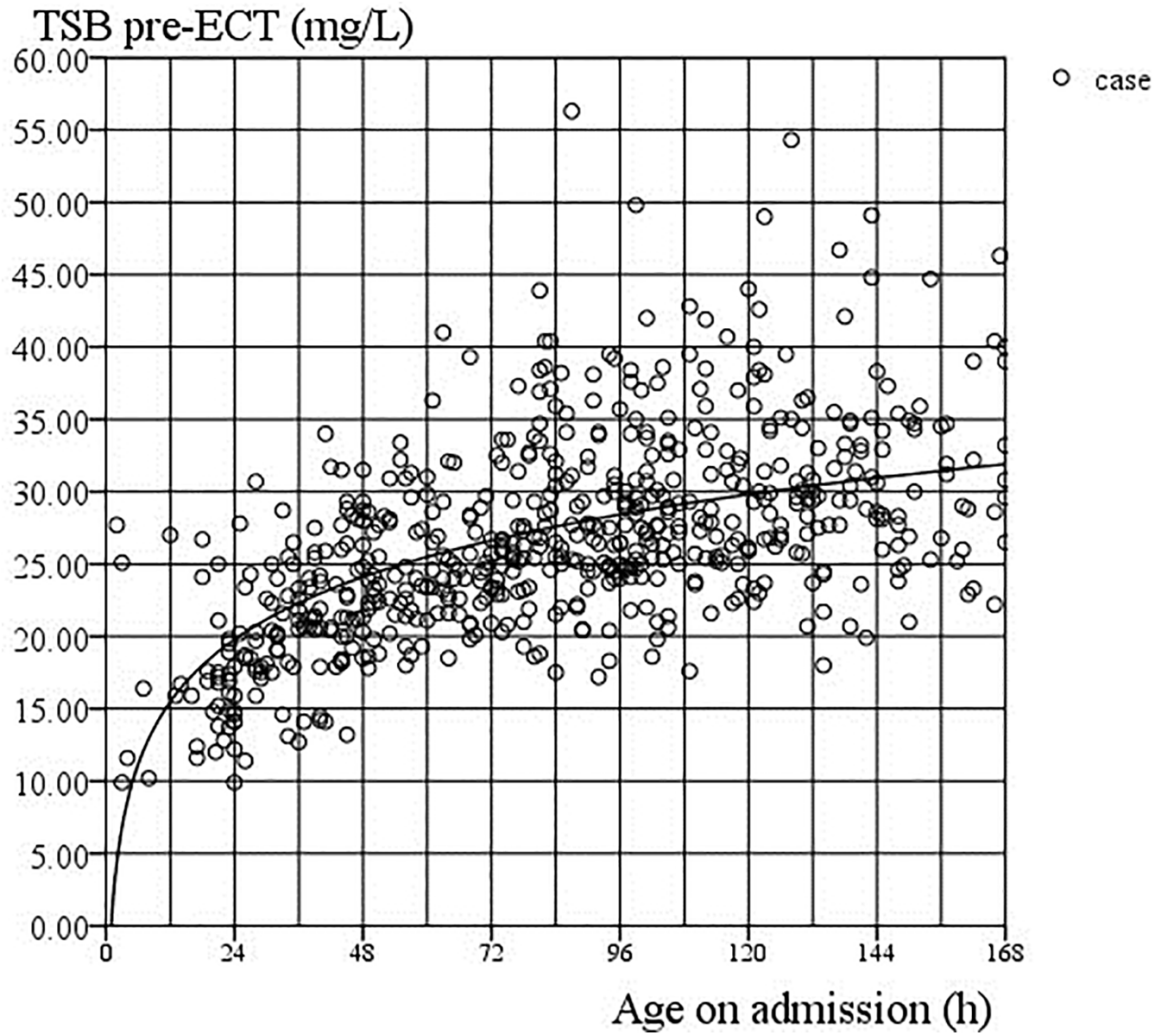
Relationship between TSB level and admission age in hours.

As shown in Table 1, TSB levels peaked within 3 to 4 days after birth. Patients had an average weight loss of almost 5.7% on average at hospital admission, of whom 93 (15.1%) lost more than 10% of their weight. There were 369 cases (60.1%) that developed ABE, of which 43 cases (7.0%) suffered a dreadful outcome. There were 272 infants evaluated with NBNA within 10 to 14 days after birth, of whom 91 (33.5%) scored below 35. Fig 1 shows the relationship between TSB level and admission age in hours: TSB (mg/dL) = 6.23×㏒(current ages) (*R*^*2*^ = 0.958, *P* <0.001). In contrast, with the ECT guidelines stipulated by the AAP in 2004, the TSB levels of most patients in this study exceeded the threshold.

**Table 1:** General characteristics (n = 614).

		n (%)		$\bar{x}$ ± SD	range
Primiparity		427 (69.5)	Gestational week (w)	39.2 ± 1.2	37~42^+6^
Male		342 (55.7)	Birth weight (g)	3302 ± 457	1800~4800
Sesarean delivery		332 (54.1)	Body weight on admission (g)	3112 ± 439	1659~4700
Pathological weight loss		93 (15.1)	Weight loss rate (%)	-5.7 ± 5	-0.25~0.2
Family history of jaundice		6 (1.0)	Age on admission (h)	83.9 ± 39.7	2~168
ABE		369 (60.1)	Onset time of jaundice (h)	35.7 ± 22.6	0~120
CT	normal	16 (17.8)	TSB_pre-ECT_ (μmol/L)	455.2 ± 118	169.1~962.4
	abnormal	74 (82.2)			
MRI	normal	6 (40.0)	Volume_exchange out_ (kg/ml)	160.5 ± 15.2	63~220.6
	abnormal	9 (60.0)			
NBNA<35		9 (33.5)	Volume_exchange in_ (kg/ml)	165.6 ± 15	90.4~224
Unfavourable outcome		43 (7.0)	Duration of ECT (min)	105 ± 23	51~240

During hospitalization, 106 patients received a cranial imaging examination; only 1 of 3 cases that underwent TCD was proven to have hydrocephalus. There were 90 cases scanned by cranial CT: 16 were normal, 65 had hydrocephalus, 9 had intracranial hemorrhage (especially in the subarachnoid space), 1 had cerebral infarction in the right lobus parietalis, 1 had dysplasia of the corpus callosum, 7 had brain parenchymal density change, 1 had ABE in combination with interhemispheric hemorrhage and cranial hematoma, and 1 had the possibility of subarachnoid hemorrhage. Among the 15 patients who received cranial magnetic resonance imaging, 6 were proven normal, whereas in 4 cases, MRIs revealed abnormal signals in the globus pallidus or brain white matter, and the rest showed hydrocephalus or cerebral hemorrhage.

### 2. Analysis of etiology

**Fig 2:**
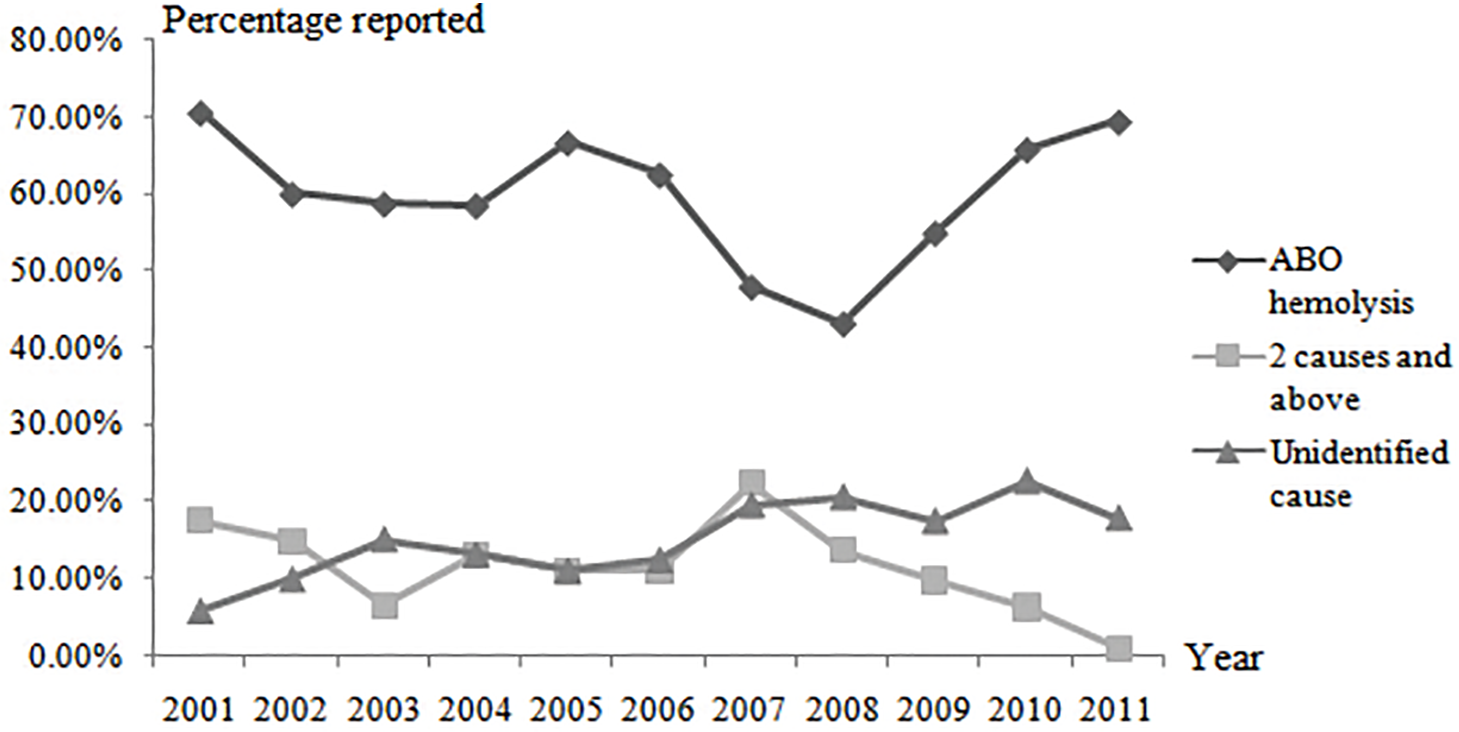
Temporal trends in causes of hyperbilirubinemia.

The major identifiable cause of severe hyperbilirubinemia is ABO incompatibility, followed by unidentified cause. The annual analysis shows that each year, 40%-70% of patients receive ECT due to ABO hemolysis; furthermore, it reveals a decrease-increase tendency. However, for those with unidentified cause, a slowly increasing trend is shown year by year (Fig 2). There were 14 cases (13.5%) with a weight loss of more than 10%. It was also shown that LGA neonates were more likely to suffer a pathological weight drop (*P* <0.05) with the highest TSB level before ECT. AGA and LGA neonates suffered a more evident weight loss than SGA neonates (*P* <0.05), but had higher pre-exchange TSB levels (*P* <0.05). However, there were no significant differences among the three types of neonates in terms of ABE level or prognosis (Table 2).

**Table 2:** Comparison of relevant information of infants with different prognoses.

		SGA^c^ (n = 48)	AGA^c^(n = 453)	LGA^c^ (n = 113)	*P*_1_^d^	*P*_2_^d^	*P*_3_^d^
Gestational age (w)		39.3 ± 1.2	39.2 ± 1.4	39.3 ± 1.2	0.609	0.834	0.580
Birth weight (g)		2498.5 ± 240.2	3220 ± 265	3974 ± 274.9	<0.001	<0.001	<0.001
Weight loss rate ^b^(%)		-3.6 ± 6.2	-5.5 ± 4.8	-7.2 ± 4.7	0.043	0.001	0.001
TSB_pre-ECT_ (μmol/L)		410.5 ± 120.2	456.6 ± 117.6	468.0 ± 115.4	0.013	0.006	0.508
ABE	no	15 (31.3%)	185 (40.8%)	46 (40.7%)	0.108		
	mild	13 (27.1%)	157 (34.7%)	44 (38.9%)			
	moderate	10 (20.8%)	64 (14.1%)	14 (12.4%)			
	severe	10 (20.8%)	47 (10.4%)	10 (8.8%)			
Unfavorable outcome^a^		2 (4.2%)	34 (7.5%)	7 (6.2%)	0.643		

^a^Unfavorable outcome: withdraw the treatment or die.

^b^Body weight loss rate = (body weight on admission–birth weight) / birth weight*100%.

^c^SGA: small for gestational age; AGA: appropriate for gestational age; LGA: large for gestational age.

^d^P1: SGA versus AGA; P2: SGA versus LGA; P3: AGA versus LGA.

### 3. Relevant adverse events attributable to exchange transfusion

Isovolumetric, double-volume fresh blood was used for ECT. As summarized in Table 3, the leading complication attributable to ECT was a biochemical abnormality, especially thrombocytopenia (54.6%) and hyperglycemia (42.8%), followed by metabolic acidosis (31.1%) and hypokalemia (31.6%). There was not a strong correlation between the incidence of complications and extent of ABE (*P* >0.05). The incidence of severe complications was 7%, with apnea predominating among them, and NEC in second place. After ECT, all neonates could suffer dreadful adverse effects, and with exacerbation of ABE, the incidence of severe complications rose evidently (*P* <0.001). No ECT-related mortality was observed in this study.

**Table 3:** Adverse events secondary to exchange transfusion.

Adverse events	Without ABE (*n* = 24)	Mild ABE (*n* = 213)	Moderate ABE (*n* = 88)	Severe ABE (*n* = 67)	Total(*n* = 614)	*P value*
Hyperglycemia	106(43.1%)	89 (41.8%)	34 (38.6%)	34(50.7%)	263(42.8%)	0.485
Thrombocytopenia	140(56.9%)	114(53.5%)	50 (56.8%)	31(46.3%)	335(54.6%)	0.444
Metabolic acidosis	74(30.1%)	65 (30.5%)	25 (28.4%)	27(40.3%)	191(31.1%)	0.377
Hypokalemia	81(32.9%)	63 (29.6%)	31 (35.2%)	19(28.4%)	194(31.6%)	0.690
Hypocalcemia	55(22.4%)	36 (16.9%)	17 (19.3%)	9 (13.4%)	117(19.1%)	0.289
Hypomeganesemia	42(17.1%)	27 (12.7%)	12 (13.6%)	10(14.9%)	91 (14.8%)	0.601
Hyponatremia	24 (9.8%)	17 (8.0%)	5 (5.7%)	6 (9.0%)	52 (8.5%)	0.684
Apnea	2	5	5	8	20 (3.3%)	<0.001
Respiratory failure	0	1	1	4	6 (1.0%)	
Heart failure	0	2	2	3	7 (1.1%)	
NEC	2	1	2	3	8 (1.3%)	
Shock	0	0	0	1	1 (0.2%)	
DIC	1	0	0	0	1 (0.2%)	

### 4. The impact on the prognosis

As shown in Table 4, jaundiced patients due to Rh hemolysis manifested the earliest onset, with the longest hospital stay and duration of phototherapy. However, patients with jaundice due to G-6-PD deficiency and sepsis revealed a later onset time and a higher TSB level than other groups. Pronounced differences in ABE degree and prognosis were documented among different groups (*P* <0.05) (Table 4). After Arun Babu T *et al*[10] followed-up with 66 term newborns with hyperbilirubinemia within 7 days after birth (bilirubin >15 mg/dL) for 6 months, it was discovered that with the rise in TSB levels, neurodevelopmental impairment increased accordingly. Rh hemolysis, jaundice occurring within two days of life and peak serum bilirubin levels >22 mg/dl are 3 independent risk factors leading to neurodevelopmental impairment for hyperbilirubinemic neonates. In summary, the icterogenic cause, onset time and pre-exchange TSB levels have an effect on the outcome. Table 5 indicates that there is a shared chance for neonates with severe hyperbilirubinemia occurring within 7 days of life to suffer severe ABE and an unfavorable outcome. Further analysis showed that pre-exchange TSB levels were significantly different in 4 different timescales (*P* <0.001); the pre-exchange TSB levels were as follows: 421.9 ± 116.8 μmol/L, 479.8 ± 109.7 μmol/L, 493 ± 118.8 μmol/L, and 502.1 ± 98.9 μmol/L. It was also revealed that jaundice peaked within 3 to 4 days of life. However, there were no significant differences in ABE degree or prognosis among the four groups. As shown in Table 5, the severity of ABE among the 5 groups varied accordingly with the increase in TSB levels (*P* <0.001), but there were no significant differences between any two groups. This means that once the pre-exchange TSB levels exceeded a certain range, ABE worsened. We did not find any significant differences in outcomes among the 5 groups (*P* <0.05). As suggested by further investigation, setting the cut-off point at 340 μmol/L showed no significant differences in ABE incidence, degree or prognosis between the 2 groups, while setting the cut-off point at 425 μmol/L showed the ABE incidence, degree and outcomes to be markedly different between the 2 groups. Additionally, the incidence of unfavorable outcomes in the TSB >425 μmol/L group was 2.08 times that of the TSB <425 μmol/L group. Setting the cut-off point at 510 μmol/L, there were still significant differences between 2 groups, and the incidence of a poor prognosis in the TSB >510 μmol/L group was 2.83 times that of the TSB <510 μmol/L group (Table 5).

**Table 4:** Comparison of the degree of ABE and prognosis among infants with different causes.

		ABO hemolysis(n = 368)	Rh hemolysis (n = 37)	G-6-PD deficiency (n = 18)	Cranial hematoma (n = 20)	Sepsis (n = 6)	Cause ≧2 (n = 62)	Unidetified cause(n = 103)	*P value*
Jaundice onset time (h)		32.3 ± 21	20.7 ± 18.3	56.1 ± 22.2	41.8 ± 19.9	67.3±18.0	37.4 ± 20.3	45.6 ± 25.6	<0.001
TSB level(μmol/L)		440.7 ± 109.7	407.4±127.8	568.5±169.3	452.0±84.4	539.5 ± 96.9	489.9±152.3	479.2 ± 92.2	<0.001
Time of phototherapy(h)		70.9 ± 27.5	86.2 ± 26.2	78.6 ± 34.4	81.7 ± 30.7	64±25.7	77 ± 29.2	72.9 ± 26.8	0.001
Time in hospital(h)		8.6 ± 3.9	9.5 ± 4.5	8.5 ± 5.2	8.6 ± 3.3	7.3±3.9	8.7 ± 4.5	7.9 ± 3.7	0.529
ABE	No	171(46.5%)	5(13.5%)	1(5.6%)	5(25.0%)	2(33.3%)	21(33.9%)	41(39.8%)	<0.001
	Mild	127(34.5%)	12(32.4%)	10(55.6%)	8(40.0%)	3(50.0%)	16(25.8)	37(35.9)	
	Moderate	41(11.1%)	12(32.4%)	3(16.7%)	4(20.0%)	1(16.7%)	11(17.7%)	16(15.5%)	
	Severe	29(7.9%)	8(21.6%)	4(22.2%)	3(15.0%)	0(0.0%)	14(22.6%)	9(8.7%)	
Unfavourable outcome		16(4.3%)	5(13.5%)	4(22.2%)	2(10.0%)	1(16.7%)	6(9.7%)	9(8.7%)	0.02

**Table 5:** The effects of the onset time of jaundice and TSB level on the severity of ABE and prognosis.

		Without ABE(*n* = 246)	Mild ABE(*n* = 213)	ModerateABE(*n* = 88)	Severe ABE(*n* = 67)	*P*_*1*_	Poor prognosis(*n* = 43)	*P*_*2*_	*OR*	95%*CI*
Age on admission (h)	≤24	112(45.5%)	101(47.4%)	47(53.4%)	28(41.8%)	0.881	14	0.252		
	24~48	96(39.0%)	77(36.2%)	25(28.4%)	26(38.8%)		21			
	48~72	28(11.4%)	27(12.7%)	13(14.8%)	10(14.9%)		6			
	>72	10(4.1%)	8(3.8%)	3(3.4%)	3(4.5%)		2			
TSB_pre-ECT_(μmol/L)	≤255	14(5.7%)	8(3.8%)	3(3.4%)	1(1.5%)	<0.001	0	0.008		
	255~340	31(12.6%)	22(10.3%)	5 (5.7%)	4(6.0%)		5			
	340~425	76(30.9%)	56(26.3%)	21(23.9%)	8(11.9%)		6			
	425~510	80(32.5%)	72(33.8%)	31(35.2%)	17(25.4%)		11			
	>510	45(18.3%)	55(25.8%)	28(31.8%)	37(55.2%)		21			
TSB_pre-ECT_> 340 μmol/L	No	45(18.3%)	30(14.1%)	8 (9.1%)	5(7.5%)	0.053	5	0.6	1.293	0.494~3.379
	Yes	201(81.7%)	183(85.9%)	80(90.9%)	62(92.5%)		38			
TSB_pre-ECT_> 425 μmol/L	No	121(49.2%)	86(40.4%)	29(33.0%)	13(19.4%)	<0.001	11	0.038	2.079	1.027~4.028
	Yes	125(50.8%)	127(59.6%)	59(67.0%)	54(80.6%)		32			
TSB_pre-ECT_> 510 μmol/L	No	201(81.7%)	158(74.2%)	60(68.2%)	30(44.8%)	<0.001	22	0.001	2.83	1.512~5.299
	Yes	45(18.3%)	55(25.8%)	28(31.8%)	37(55.2%)		21			

### 5. Analysis of clinical materials of infants with different prognoses

The pre-exchange TSB level of patients with an unfavorable outcome was evidently higher than in patients with a promising outcome. The admission time of the former group was markedly later than that of the latter group (*P <*0.05). ABO hemolysis, G-6-PD deficiency and cesarean delivery posed a more favorable prognosis (*P <*0.05) (Table 6). In this study, 306 cases received the BAEP test, and 94 of these cases manifested an abnormal BAEP, of which 5 cases suffered a bad prognosis. There were 212 cases identified as normal without any unfavorable outcomes. A c*hi-squared* test indicated that patients with an abnormal BAEP may have a higher incidence of a poor prognosis than those with normal results (*P =* 0.003, *OR =* 1.056, 95% *CI*: 1.007–1.108). Of the 246 cases that underwent a B/A ratio test, 52 cases had a B/A ratio >1, of which 7 suffered a poor prognosis. There were 194 cases with a B/A ratio ≤1, of which 6 cases had a poor prognosis. A c*hi-squared* test indicated that patients with a B/A ratio >1 had a higher incidence of an unfavorable outcome than those with a B/A ratio ≤1 (*P =* 0.008, *OR =* 4.874, 95% *CI*: 1.562–15.208).

**Table 6:** Comparison of relevant information of infants with different prognoses (n = 614).

	Number				$\bar{x}$±SD		
	Good prognosis n = 571	Poor prognosis n = 43	*P value*		Good prognosis n = 571	Poor prognosis n = 43	*P* *value*
Male	315	27	0.332	Birth weight (g)	3305.6 ± 463.5	3254 ± 365	0.658
ABO hemolysis	352	16	0.002	Gestational age (w)	39.2 ± 1.4	39.2 ± 1.3	0.934
Rh hemolysis	32	5	0.17	Age on admission (h)	82.6 ± 39.3	100.8 ± 42.6	<0.001
G-6-PD deficiency	14	4	0.031	Onset time (h)	35.3 ± 22.5	40.8 ± 23.4	0.09
Comorbid disease	56	6	0.426	TSB peak level before ECT (μmol/L)	449.3 ± 112.9	533.3 ± 152.3	0.001
Sepsis	5	1	0.354	TSB clearance rate (%)	54.1 ± 9.7	51.7 ± 14.8	0.173
Cranial hematoma	18	2	0.644				
Unidentifie cause	94	9	0.45	Weight loss rate (%)	-5.7 ± 5	-6.1 ± 5.3	0.575
Cesarean delivery	318	14	0.003				

## Discussion

Extreme neonatal hyperbilirubinemia, by no means uncommon, the results primarily from maternofetal blood group isoimmunization, especially ABO hemolysis. There were 368 extremely hyperbilirubinemic neonates (59.9%) in this study documented with ABO hemolysis. According to the annual analysis, 40%-70% of patients received ECT due to ABO hemolysis. Unfortunately, in this study, 103 cases (16.8%) are listed as “idiopathic”; so far, the etiology of these cases remains controversial. Christensen RD *et al* [11] believed that hyperbilirubinemia of TSB >25 mg/dl with unknown etiology was caused by hemolytic disease. However, this study showed that 14 cases (13.5%) with unknown cause had a weight loss rate of more than 10%. Macdonald PD *et al* [12] investigated 937 newborns with body weight ≥2500 g and discovered that the mean weight loss rate of breast-fed newborns was 6.6% and that the lowest weight occurred at 2.7 days of life. Flaherman VJ *et al* [13] investigated 47,687 newborns of GA ≥36 weeks, fed exclusively by breastfeeding or mixed feeding, and indicated that the weight loss rate was 6.3 ± 3.5%, of which 4580 cases (9.6%) suffered a weight loss rate of more than 10%. There were 2670 cases that lost more than 5% body weight within 24 hours after birth, of which 782 cases (29.0%) developed pathological weight loss (*P* <0.05). According to the multi-factorial analysis, irrespective of the gestational age, delivery mode, nation of the mother and labor hospital, a weight loss rate ≥5% within 24 hours after birth carries a direct risk for newborns to develop pathological weight loss in the future (*OR =* 4.06, 95% *CI*: 3.69–4.46). Chang RJ *et al* [14] also reported that a weight loss rate exceeding 8% after 48 h or a weight loss rate exceeding 11% after 72 h can be taken as ominous predictors of severe hyperbilirubinemia in the future, with negative predictive values of 77.7% and 76.8%, respectively. It was also revealed in this study that AGA and LGA neonates suffered a more evident weight loss, with a higher pre-exchange TSB level, which probably involved intake insufficiency, since AGA and LGA neonates require more energy and fluid than SGA neonates. Energy insufficiency and dehydration accelerate enterohepatic circulation, resulting in the exacerbation of jaundice. Therefore, for early-stage newborns, especially LGA neonates, improved lactation support is an effective way to reduce the risk of hyperbilirubinemia. In the American Academy of Pediatrics clinical practice guidelines (2004) [7], it is mentioned that the East Asian population, including the population in mainland China, Japan, Korea, Hong Kong, and Taiwan, is a major risk factor for severe hyperbilirubinemia. In addition to the higher incidence of hemolytic disease noted above (e.g., ABO hemolysis, G-6-PD deficiency), it has also been hinted that genetic factors might play a certain role in this process. However, since this study was limited by insufficient medical resources during a long period of 11 years (from 2001 to 2011), it was hard to detect the variation of the *UGT1A1* gene as a regular neonatal screening. In recent years, more and more reports have cast light on the relationship between *UGT1A1* polymorphism and severe hyperbilirubinemia, recommending that it is crucial to explore the pathogenesis in the gene level. From 2012 to 2014, a retrospective study about *UGT1A1* gene polymorphism among unconjugated hyperbilirubinemic full-term neonates in the Chongqing area was performed and published [15]. In this clinical study, 330 term neonates with unconjugated hyperbilirubinemia were enrolled and divided into two groups (210 neonates with identified cause and 120 neonates with unknown cause). It was revealed that among those with unknown cause, the total variation rate, homozygous variation rate and heterozygous variation rate at 211G>A of the *UGT1A1* gene was 49.2%, 11.7% and 37.5%, respectively, significantly higher than those with identified cause, which was 38.6%, 6.2% and 32.4%, respectively, (P<0.001). *Logistic regression* indicated the odds ratios of neonates who carried a 211G>A mutation of the *UGT1A1* gene associated with the development of unconjugated hyperbilirubinemia and severe jaundice in full-term neonates were 1.54 (3.083–8.108) and 2.64 (1.278–4.508), respectively. From March 2016 to April 2016, a prospective study was implemented to further prove this standpoint (the manuscript is under review). A total of 219 term and late pre-term infants were enrolled, and umbilical cord blood was tested to determine *UGT1A1* gene polymorphism (including the promoter region and exon 1 sequence). In this study, the total 211 G>A mutation incidences among hyperbilirubinemic infants and non-hyperbilirubinemic infants were 51.7% and 30.0%, respectively. *Logistic regression* indicated that the OR (95% CI) of 211 G>A mutation associated with high-risk TCB levels within 1–3 days, 4–7 days and phototherapy risk was 4.49 (2.42–8.34), 4.36 (2.36–8.04) and 2.50 (1.13–5.52), respectively. Therefore, the *UGT1A1* 211 G>A mutation is a risk factor for neonatal hyperbilirubinemia among both term and late pre-term infants in Chongqing, China, which could be a common cause of severe jaundice with unknown cause.

ECT is one of the emergency interventions for extreme neonatal hyperbilirubinemia. This therapy has been implemented for many years in the world, and its timeliness and efficacy in reducing TSB load and preventing ABE have been widely acknowledged. However, some previous studies have mentioned that ECT poses a threat of internal disorder, even resulting in respiratory failure or cardiac arrest. No ECT-related mortality was documented in this study. The major complication secondary to ECT was a biochemical abnormality, most recovering within 2 to 3 days after the ECT procedure. Davutoğlu M *et al* [16] analyzed 79 patients, revealing that the most common adverse events were thrombocytopenia and convulsion. Sakha SH *et al* [17] investigated 150 newborns undergoing ECT, indicating that most of the adverse events induced by ECT were asymptomatic and reversible. In this study, 43 patients (7%) suffered severe adverse events, and apnea (3.3%) was predominant. This finding probably involves limited compensation of the neonatal respiratory system, respiratory muscle fatigue and suspected ABE. Next, was NEC (1.3%), which resulted from intestinal ischemia and necrosis because during the ECT procedure, the counter-pressure produced by the portal vein system blocks blood flow. It has been revealed that ECT-related adverse events are inevitable, so the best way to reduce the occurrence is to avoid ECT through close follow-up after discharge and timely intervention of neonatal hyperbilirubinemia.

At present, a clinician determines whether or not the neonate needs ECT according to the TSB level, referring to the Jaundice Interference Manual. Duman N *et al* [18] thought that newborns had better tolerance to bilirubin neurotoxicity without the suggested risk factors, therefore, they proposed to modify the existing ECT guidelines by properly enhancing the bilirubin threshold. After Ding Guofang *et al* [19] studied 875 normal one-month newborns, it was discovered that TSB levels were different in the neonates born in different regions and seasons. It was suggested that for normal, full-term neonates, the diagnostic standard should exceed the existing criterion; meanwhile, the intervention should be reinforced accordingly. Most of the existing risk factors were included in this study (e.g., our country is located in East Asia, over 65% of newborn patients suffer hemolytic disease), and most TSB levels were far above the recommended exchange transfusion thresholds as per the AAP. Nevertheless, after emergency treatment, only 43 cases suffered a poor prognosis, which indicates that the neonates in Southwestern China have a better tolerance to bilirubin neurotoxicity.

Although ECT therapy can remove bilirubin, antibodies and sensitized erythrocytes as well as prevent ABE, ECT still cannot improve outcomes if patients are not managed promptly. Most of the 614 cases enrolled in this study had a favorable outcome, especially those with ABO hemolysis, G-6-PD deficiency or cesarean delivery. This may be attributable to comprehensive prenatal examinations, timely management, and more attention paid by doctors. It was shown that the onset time of patients with ABO hemolysis was 32.3 ± 21 h, which was earlier than that of the group as a whole (35.3 ± 22.5 h) (*P =* 0.026), so those with ABO hemolysis need more prompt intervention. In 1980, the Blood Research Institute of China Academy of Medical Sciences conducted a survey of 923 newborns in Jianyang, Sichuan for the first time, showing that the rate of occurrence of G-6-PD deficiency for newborns was 4.6%. After the implementation of a minimum methemoglobin oxidation experiment and several preventive approaches, the incidence of G-6-PD deficiency was reduced by approximately 50%; meanwhile, the incidence of induced kernicterus decreased from 12.4% to 0.9% [20]. Lam *et al* [21] reported that in the 1970s, 45% of jaundiced newborns needed ECT, 8.6% of whom developed kernicterus, and the mortality rate caused by kernicterus was 20%. Since the G-6-PD deficiency screening project for newborns was launched in 1981, only 1.6% of jaundiced newborns needed ECT, and none of them developed kernicterus. It is supposed that the popularization of G-6-PD deficiency screening gradually shed light on the subject, leading people to consider several prenatal measures and reminding clinicians to pay more attention to hyperbilirubinemic neonates induced by G-6-PD deficiency.

Unfortunately, there were still 43 patients who suffered unfavorable outcomes, which might be multi-factorial. It was also found that the higher the TSB level, the higher the likelihood that the patient suffered a poor prognosis. This was especially true when the TSB level exceeded 510 μmol/L, as the incidence of an unfavorable prognosis approximately tripled. However, when the TSB level was 255–340 μmol/L, there were still patients who suffered a poor prognosis, indicating that the TSB level was not the unique factor determining the outcome. The onset time might also have contributed to the prognosis. Arun Babu T *et al* [10] reported that onset time of jaundice within 48 hours after birth was one independent risk factor for neuro-developmental impairment. It was shown that there was a difference in pre-exchange TSB peak values in different onset timescales, but there were no differences documented in severity or prognosis. This might suggest that the parents could not provide an accurate onset time and follow-up information. The peak TSB level generally occurs within 3 to 4 days after birth, whereas many newborns were admitted to the hospital more than 4 days after birth, missing the optimal time for treatment. ECT could reduce the TSB level, but could not reverse the brain injury. Sgro M *et al* [22] reported that approximately 30% of extremely severe jaundiced neonates suffered acute neurological injury if the TSB level was higher than 550 μmol/L, whereas neonates presenting severe jaundice within 2 days of life or who were treated with ECT were at a higher risk for ABE (*OR* = 3.332 and 2.572, respectively). Similar findings were also observed in this study: the extremely severe patients with TSB levels higher than 510 μmol/L were at a higher risk for an unfavorable prognosis (*OR* = 2.83). After Zhuang Yan *et al* [23] investigated 967 newborns suffering from hyperbilirubinemia, it was shown that a total bilirubin/albumin ratio (B/A) >1 and acidosis were high risk factors of abnormal BAEP. Saluja S *et al* [24] noted that if newborns with severe jaundice exhibit hearing impairment (auditory neuropathy), bilirubin-induced neurotoxicity should be highly considered. In this study, occurrence of a poor prognosis in those with abnormal BAEP and with B/A >1 was significantly higher. Cranial MRI is indispensable for judging the severity and prognosis of hyperbilirubinemia. Campistol J *et al* [25] reported that the most typical imaging change of neonatal ABE was the increased signal intensity in the globus pallidus in a T1 weighted image. After Katar S *et al* [26] analyzed 21 hyperbilirubinemic term newborns undergoing ECT, it was believed that cranial MRI played an essential role in the diagnosis of ABE; however, MRI appearance was not always consistent with the clinical manifestation. That is why only 4 of 15 patients who received and MRI in this study were proven to have ABE.

## Conclusion

The etiology, peak TSB level before ECT and time of ECT have a significant impact on the outcome of neonatal severe hyperbilirubinemia. ABO incompatibility is the most common cause of extreme neonatal hyperbilirubinemia. Pathological weight loss could be involved in the development of extreme hyperbilirubinemia with an unidentified cause.

## Supporting information

S1 File Ethics approval. (ZIP)

S2 File Underlying data. (XLSX)
