# Supplementary figures and images for: Report about term infants with severe hyperbilirubinemia undergoing exchange transfusion in Southwestern China during an 11-year period, from 2001 to 2011
Source: PLoS One. 2017 Jun 29;12(6):e0179550. doi: 10.1371/journal.pone.0179550 (PMC5491324; doi:10.1371/journal.pone.0179550)

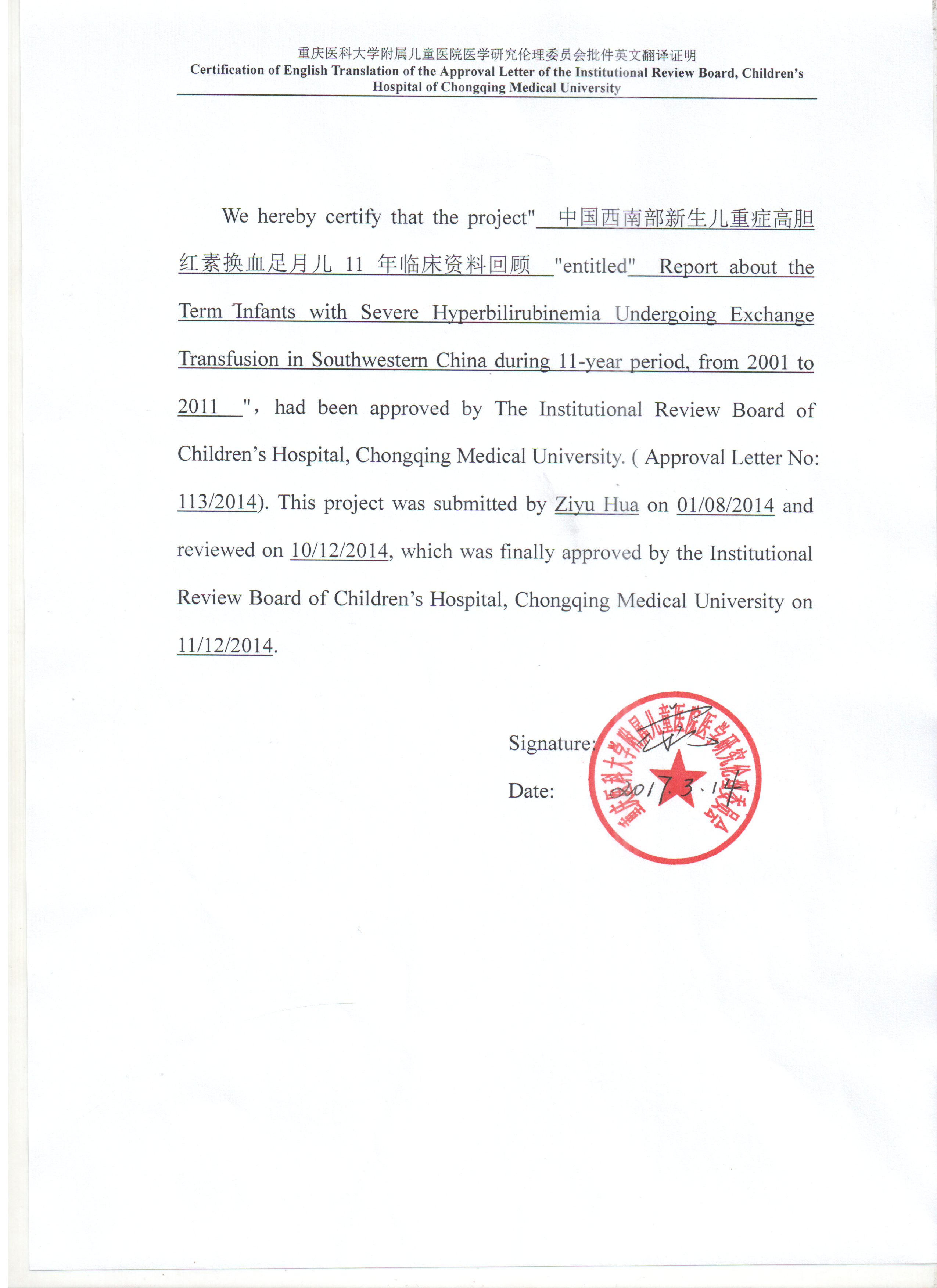

Supplement: S1 File — (ZIP) [file pone.0179550.s001.zip › ethics approval/certification of English translation of ethics approval.tif]

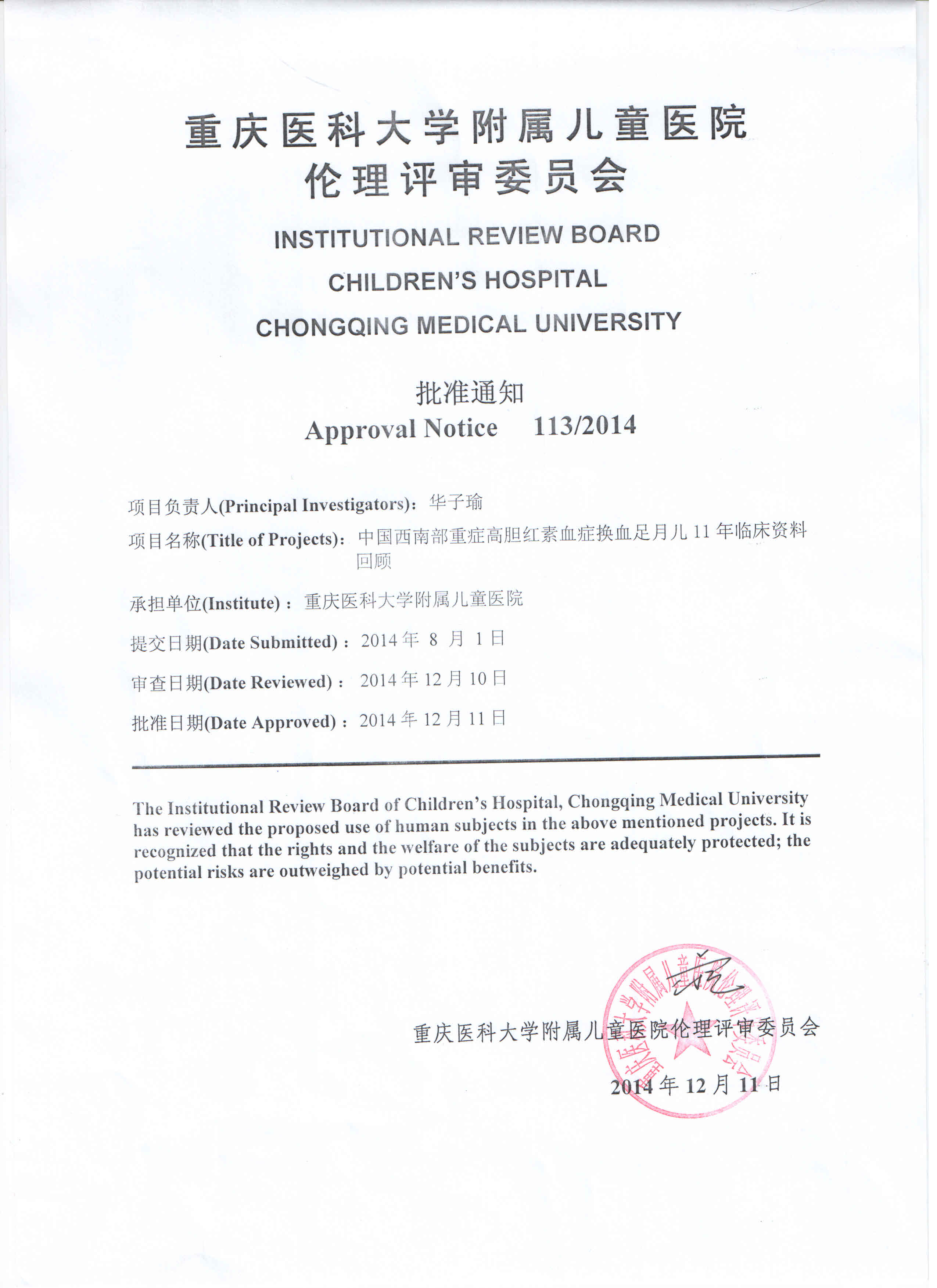

Supplement: S1 File — (ZIP) [file pone.0179550.s001.zip › ethics approval/ethics approval.tif]
